# Networking retinomorphic sensor with memristive crossbar for brain-inspired visual perception

**DOI:** 10.1093/nsr/nwaa172

**Published:** 2020-07-25

**Authors:** Shuang Wang, Chen-Yu Wang, Pengfei Wang, Cong Wang, Zhu-An Li, Chen Pan, Yitong Dai, Anyuan Gao, Chuan Liu, Jian Liu, Huafeng Yang, Xiaowei Liu, Bin Cheng, Kunji Chen, Zhenlin Wang, Kenji Watanabe, Takashi Taniguchi, Shi-Jun Liang, Feng Miao

**Affiliations:** National Laboratory of Solid State Microstructures, School of Physics, Collaborative Innovation Center of Advanced Microstructures, Nanjing University, Nanjing 210093, China; National Laboratory of Solid State Microstructures, School of Physics, Collaborative Innovation Center of Advanced Microstructures, Nanjing University, Nanjing 210093, China; National Laboratory of Solid State Microstructures, School of Physics, Collaborative Innovation Center of Advanced Microstructures, Nanjing University, Nanjing 210093, China; National Laboratory of Solid State Microstructures, School of Physics, Collaborative Innovation Center of Advanced Microstructures, Nanjing University, Nanjing 210093, China; National Laboratory of Solid State Microstructures, School of Physics, Collaborative Innovation Center of Advanced Microstructures, Nanjing University, Nanjing 210093, China; National Laboratory of Solid State Microstructures, School of Physics, Collaborative Innovation Center of Advanced Microstructures, Nanjing University, Nanjing 210093, China; National Laboratory of Solid State Microstructures, School of Physics, Collaborative Innovation Center of Advanced Microstructures, Nanjing University, Nanjing 210093, China; National Laboratory of Solid State Microstructures, School of Physics, Collaborative Innovation Center of Advanced Microstructures, Nanjing University, Nanjing 210093, China; School of Electronic Science and Engineering, Nanjing University, Nanjing 210093, China; School of Electronic Science and Engineering, Nanjing University, Nanjing 210093, China; School of Electronic Science and Engineering, Nanjing University, Nanjing 210093, China; National Laboratory of Solid State Microstructures, School of Physics, Collaborative Innovation Center of Advanced Microstructures, Nanjing University, Nanjing 210093, China; National Laboratory of Solid State Microstructures, School of Physics, Collaborative Innovation Center of Advanced Microstructures, Nanjing University, Nanjing 210093, China; School of Electronic Science and Engineering, Nanjing University, Nanjing 210093, China; National Laboratory of Solid State Microstructures, School of Physics, Collaborative Innovation Center of Advanced Microstructures, Nanjing University, Nanjing 210093, China; National Institute for Materials Science, Tsukuba 305-0044, Japan; National Institute for Materials Science, Tsukuba 305-0044, Japan; National Laboratory of Solid State Microstructures, School of Physics, Collaborative Innovation Center of Advanced Microstructures, Nanjing University, Nanjing 210093, China; National Laboratory of Solid State Microstructures, School of Physics, Collaborative Innovation Center of Advanced Microstructures, Nanjing University, Nanjing 210093, China

**Keywords:** van der Waals heterostructure, retinomorphic sensor, memristive crossbar, brain-inspired visual perception, neuromorphic computing

## Abstract

Compared to human vision, conventional machine vision composed of an image sensor and processor suffers from high latency and large power consumption due to physically separated image sensing and processing. A neuromorphic vision system with brain-inspired visual perception provides a promising solution to the problem. Here we propose and demonstrate a prototype neuromorphic vision system by networking a retinomorphic sensor with a memristive crossbar. We fabricate the retinomorphic sensor by using WSe_2_/h-BN/Al_2_O_3_ van der Waals heterostructures with gate-tunable photoresponses, to closely mimic the human retinal capabilities in simultaneously sensing and processing images. We then network the sensor with a large-scale Pt/Ta/HfO_2_/Ta one-transistor-one-resistor (1T1R) memristive crossbar, which plays a similar role to the visual cortex in the human brain. The realized neuromorphic vision system allows for fast letter recognition and object tracking, indicating the capabilities of image sensing, processing and recognition in the full analog regime. Our work suggests that such a neuromorphic vision system may open up unprecedented opportunities in future visual perception applications.

## Teaser

Networking the van der Waals heterostructure sensor array with the memristive crossbar allows for neuromorphic vision system, which shows promise in pattern recognition and object tracking applications.

## INTRODUCTION

The human vision system (HVS) is mainly composed of the retina and visual cortex of the brain. It shows a powerful capability in visual perception while consuming far less than 20 W of power. Such features of the HVS strongly rely on the simultaneous sensing and early processing of visual information in the retina and parallel visual cognition in the visual cortex [1,2]. Inspired by the HVS, artificial vision systems (also known as machine vision) were developed to achieve capabilities similar to visual perception [3]. However, in conventional artificial vision systems, high redundant visual data throughput and physical separation of sensing and processing lead to high latency and large power consumption. Moreover, processing the non-structural visual data that involve heavy matrix multiplications to realize pattern recognition further increases the latency and energy consumption due to the well-known memory wall in the von Neumann architecture, which renders great challenges in practical applications, especially with the explosive growth of visual information every day. Thus, it is highly desirable to develop neuromorphic vision systems through highly precise emulation of the HVS to solve such challenges [4].

Prior works have shown that the memristive crossbar is one of the most promising neuromorphic architectures [5–9]. It holds great promise in processing image and video data with many advantages such as ultra-low power consumption and parallel computing by exploiting the physical attributes of the crossbar [5,8,10–14]. Besides, an artificial neural network (ANN) implemented on the memristive crossbar enables the capability of pattern recognition and resembles the processes of visual cognition by the HVS [15,16]. To develop a memristive crossbar-based neuromorphic vision system, one needs to network it with a retinomorphic sensor which is able to closely mimic the physical organizations and biological functions of the retina. Although previous proposals allow for simultaneously achieving sensing and early processing of the visual information based on conventional materials, they fail to mimic the hierarchical organization of the retina [17–21]. Interestingly, recent efforts have shown the potential of two-dimensional (2D) materials in neuromorphic computing [22–33] and in-sensor processing [17,18,27–29,32–34], due to their gate-tunable electronic and optoelectronic properties. Stacking of distinct 2D materials would form a diversity of van der Waals (vdW) heterostructures with richer optoelectronic properties [35–39] for various applications such as retinomorphic sensors [40].

In this work, we propose a neuromorphic vision system composed of a retinomorphic sensor and a memristive crossbar. We fabricate the retinomorphic sensor based on WSe_2_/h-BN/Al_2_O_3_ vdW heterostructure to emulate the retinal function of simultaneously sensing and processing an image. The image pre-processing occurring in the sensor enables the drastic reduction of the subsequent computational workload in the neural network implemented with the memristive crossbar. Furthermore, we network the sensor with a large-scale Pt/Ta/HfO_2_/Ta 1T1R memristive crossbar to realize distinct applications, e.g. image recognition and object tracking. This work indicates that the proposed neuromorphic vision system is promising in real-time and low-power visual perception applications.

## RESULTS AND DISCUSSION

**Figure 1.**
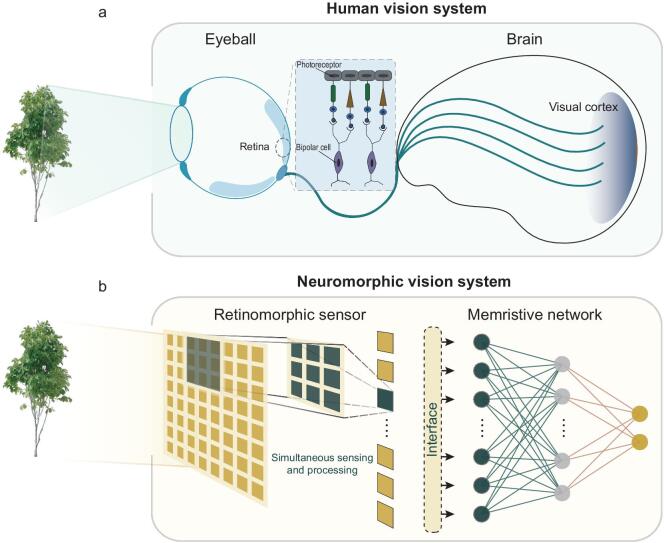
The neuromorphic vision system. The diagram schematically shows the human vision system in (a) and the neuromorphic vision system in (b), which contains a retinomorphic sensor and a memristive network.

Figure 1a shows a schematic diagram of the HVS, two primary components of which are the retina and visual cortex. The function of the retina is to sense and convert the light signals representing image information (e.g. a tree) into electrical signals through the photoreceptor. The electrical signals then quickly flow to the bipolar cell through the visual pathway and are processed to extract the key characteristics. The retained image information after early processing is eventually transmitted to the visual cortex through the optic nerve to achieve further processing and understanding of the sensed image information. With the early processing occurring in the retina, the redundant information irrelevant to the image can be discarded and consequently the understanding of the image is accelerated in the visual cortex, which has inspired a hierarchical model of object recognition that has been widely used in computer vision [41,42]. By closely mimicking the HVS, we propose a neuromorphic vision system composed of a retinomorphic sensor and a memristive network, as schematically shown in Fig. 1b. We built the retinomorphic sensor by assembling vdW heterostructure devices and implemented the memristive network by fabricating a large-scale memristive crossbar, which will be later discussed in detail. With this proposed technology, we can use the retinomorphic sensor to emulate the hierarchical organization and biological functions of the retina and avoid the physical separation between sensing and processing that is seen in conventional machine vision. In this way, the burden imposed on the limited transmission bandwidth in conventional machine vision can be released and the resulting high latency is minimized. The advantage of early processing in the retinomorphic sensor is not limited to high-speed transmission, but it also enables drastic reduction of the processing load of the image in the networked memristive crossbar, which emulates the function of the visual cortex of the human brain. Using the neuromorphic crossbar to replace the conventional processor based on the von Neumann architecture, the sensed analog information can be directly processed without analog–digital conversion. Moreover, the frequent data movement between processing and memory unit, as seen in conventional machine vision, can be eliminated, giving rise to low latency and low power consumption.

**Figure 2.**
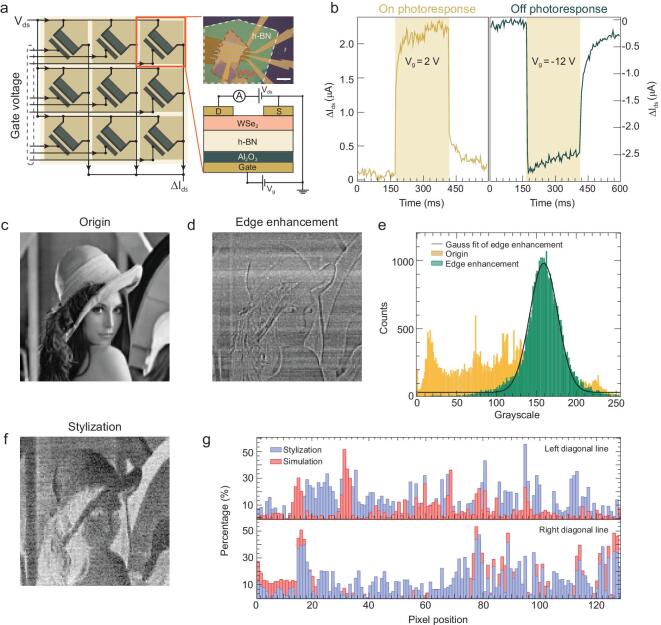
Gate-tunable photoresponse of the retinomorphic sensor and its applications in image processing. (a) 3 × 3 retinomorphic sensor based on WSe_2_/h-BN/Al_2_O_3_ vdW heterostructure device as shown in the optical image. The scale bar is 15 μm. (b) The corresponding On or Off photoresponse of the heterostructure device at V_ds _= 0.15 V. (c) The original Lenna image. The processed image by edge enhancement (d) and stylization (f) implemented with the retinomorphic sensor. (e) Grayscale distribution of the original Lenna (orange) and processed one with the edge enhancement (green). The experimental data are fitted with a Gaussian function (black solid line). (g) The comparison between the experimental results (blue, stylization in f) and the simulation of grayscales distributed in the two diagonal lines (left and right) of the Lenna image.

We use vdW heterostructure to fabricate the retinomorphic sensor which emulates the hierarchical structure and biological function of the retina in a natural way. Figure 2a schematically shows a 3 × 3 phototransistor array used as the retinomorphic sensor, in which each vdW heterostructure device serves as a pixel. To fabricate the vdW heterostructure device, we mechanically exfoliated WSe_2_ (∼20 nm) and h-BN (∼35 nm) flakes and then transferred them onto the Al_2_O_3_ dielectric layer (8 nm) in a consecutive way. The fabrication details are provided in the Methods section.

We then characterized electrical behaviors of the vdW devices under the conditions of dark and light illumination. Under the light illumination, the devices exhibit distinct optoelectronic characteristics under different polarities of back-gate voltage, with results shown in Fig. 2b. At the positive gate voltage (e.g. V_g_ = 2 V), the device shows an On photoresponse, while applying negative gate voltage (e.g. V_g_ = −12 V) results in an Off photoresponse. Current-voltage characteristics at different gate voltages and field effect curves are shown in Supplementary Figs 1 and 2, respectively. The Off photoresponse is related to the light induced charge transfer and resulting electrical field screening effect of the gate voltage [43–45], which is totally different from the negative photoconductivity phenomenon reported in ReS_2_/h-BN/MoS_2_ heterostructure. These distinct photoresponses of the vdW device resemble the light-stimulated biological response of the bipolar cell in the retina, which is a key component for processing sensed information in the visual pathway [46], and the timescale of photoresponse is comparable to the retina (Supplementary Fig. 3) [47]. By assembling nine vdW heterostructure devices into an array as shown in Fig. 2a, we are able to process the visual information on the pixel level. The processed image is represented as the variation of output current (ΔI_ds_), which is a summation of current in all individual vdW devices of the retinomorphic sensor through Ohm's law and Kirchhoff's current law. Note that we used the mechanically exfoliated 2D materials flakes at the proof-of-concept stage. However, large-area 2D materials can be used to achieve vertical integration in the future, since previous works have demonstrated successful synthesis of wafer-scale single-crystal 2D materials [48–50].

With separate control of gate voltage, we use the retinomorphic sensor to implement different convolution kernels to process the Lenna image (Fig. 2c). The grayscale information of the Lenna image was first converted into a sequence of voltage signals. Subsequently, the voltage signals were used to control the light intensity of the laser through a voltage relay to scan the image line by line. The varying light intensity shed on the sensor causes the change of ΔI_ds_ and represents the image processing. Eventually, the processed image was reconstructed by using the measured ΔI_ds_. Figure 2d presents the processed Lenna image by edge enhancement. Apparently, the profiles of the processed image are enhanced over that of the original image. To mathematically confirm the validity of this kernel, we counted grayscales of the original (orange) and the processed (green) images and presented the distribution of counts versus grayscales in the histogram, with results shown in Fig. 2e. Compared to the broad grayscales distribution in the original image, the grayscales of the processed image exhibit a very narrow distribution, which follows a Gaussian distribution. For comparison, we also carried out corresponding simulations, which are in good agreement with the experimental results (Supplementary Fig. 4).

In addition to the edge enhancement, we also implemented the image stylization kernel with the sensor to process the Lenna image in a different manner. As shown in Fig. 2f, the processed image is consistent with the simulation results. Similarly, we evaluated the validity of this kernel by making a comparison between the processed (or simulated) image and the inverted original image (Supplementary Fig. 5). The normalized error is defined as (*G_Exp__/__Sim_* − *G_i_*)/255×100%, where *G_Exp__/__Sim_* and *G_i_* respectively represent the grayscale in the left and right diagonals of the experimental (or simulation) image and the inverted original image. Figure 2g shows the experimental and simulation errors versus pixel positions along the left and right diagonals. Notably, the experimental error is nearly identical to the error of simulation and is <20% in the majority of cases. We also calculated the structural similarity (SSIM) to comprehensively characterize the similarity between the experimental and the simulation images by taking light intensity, contrast and structural information into account. The SSIM parameter varies from 0 to 1 and is widely used in computing vision for evaluating the similarity of two images. The SSIM of the images by the edge enhancement and stylization is 0.59 and 0.38, respectively. Although the SSIM is not large enough due to the non-uniformity of device performance, it is still an indication that the retinomorphic sensor may benefit intelligent Internet of Things applications with increasing demand for the early processing of sensed visual information.

**Figure 3.**
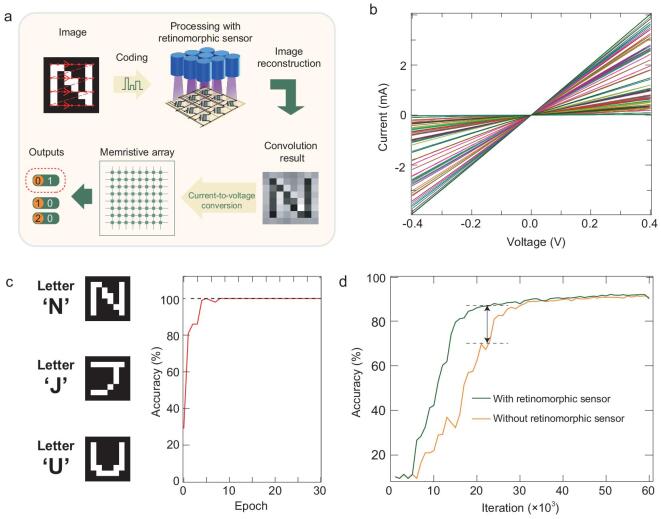
Neuromorphic vision system comprised of the retinomorphic sensor and the memristive crossbar. (a) The flow chart schematically illustrates the image sensing and processing by the retinomorphic sensor and image recognition by the memristive crossbar. (b) The linear I-V performance of the memristor with different conductances. (c) The image recognition by the neuromorphic vision system. Left panel: ‘N’, ‘J’ and ‘U’ for training the memristive neural network; Right panel: Recognition accuracy. (d) Comparison of recognition accuracy with and without the retinomorphic sensor.

Networking the retinomorphic sensor with a large-scale memristive crossbar allows for realization of brain-inspired visual perception applications (Fig. 3a). In such a networked system, the memristive crossbar is integrated with the 1T1R cell to mitigate the sneak-path current issue. The fabrication details of crossbar are given in the Methods section. We characterized the fundamental I-V characteristics of the memristive device with different conductances and presented the corresponding results in Fig. 3b. The excellent linearity of the I-V curves allows for accurate analog computing on the memristive crossbar and the emulation of the function of the visual cortex in the brain. Thus, networking the retinomorphic sensor with the memristive crossbar enables us to closely mimic the biological function of the HVS and realize image sensing, processing and recognition in the full analog regime. Based on the networked system, an image can be detected and pre-processed by the sensor to remove redundant information and only retain key information. The output from the sensor is converted into voltage signals and then input into the trained memristive neural network for perception without suffering issues related to analog–digital conversion.

The brain-inspired neuromorphic vision system is very efficient in pattern recognition. To demonstrate the image recognition, we used 2100 images of (8 × 8) English alphabets ‘N’, ‘J’ and ‘U’ (left panel in Fig. 3c and Supplementary Fig. 6). No significant degradation was observed in the process of image sensing and processing (Supplementary Fig. 7), indicating the robustness of the retinomorphic devices. The recognition output is a column vector ranging from 0 to 1, as demonstrated in the bottom left of Fig. 3a. The maximum output value in the column vector corresponds to the recognized letter. The neuromorphic visual system achieves a 100% recognition accuracy (right panel in Fig. 3c). The excellent performance of the neuromorphic visual system in image recognition suggests that integrating the retinomorphic sensor and memristive crossbar may open up a new avenue for achieving highly compact and efficient intelligent machine vision.

Early processing of the image in the retinomorphic sensor of the neuromorphic vision system can accelerate image recognition in the memristive neural network, which shows an advantage in processing a large number of images. For proof-of-concept demonstration, we have used 15 000 handwritten numerals (8 × 8) derived from the Modified National Institute of Standards and Technology database as the test input. The recognition output is a 1 × 10 column vector ranging from 0 to 9. The maximum output value in the column vector corresponds to the recognized numeral. We compared the recognition accuracy with and without the retinomorphic sensor, with results shown in Supplementary Fig. 8. Although the early processing of the handwritten numerals in the retinomorphic sensor leads to a negligible improvement of the recognition accuracy, it remains effective in accelerating convergence speed of the recognition with the retinomorphic sensor, which is not obvious due to the limited size of the fabricated memristive crossbar and the pixel-to-pixel variation of the retinomorphic sensor. We show that expanding the memristive neural network scale drastically speeds up the convergence of the numeral recognition (Fig. 3d), as compared to that without the retinomorphic sensor. Note that further optimization of fabrication processes, and expanding the retinomorphic sensor array and the memristor crossbar array, are expected to considerably improve the recognition accuracy and convergence rate.

**Figure 4.**
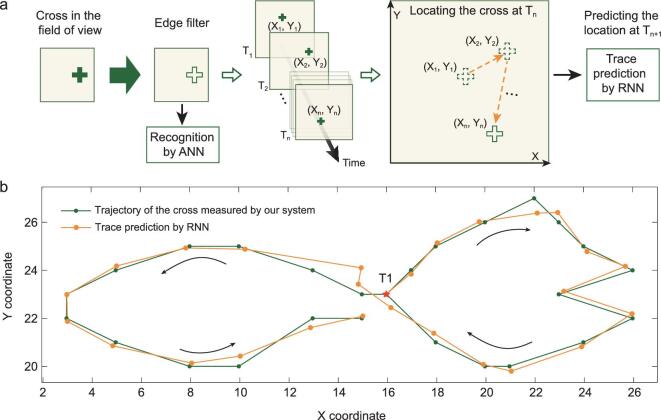
Object tracking of neuromorphic vision system. (a) The flow chart of the object tracking based on the neuromorphic vision system. The box represents the field of view defined by the retinomorphic sensor. By using the sensor, the cross edge in the field of view is extracted as its key feature. Once the cross is recognized by the ANN, the position information (X_n_, Y_n_) of the cross at T_n_ is input into a trained RNN to achieve the object tracking. (b) The trajectory of the cross measured by the neuromorphic vision system (green line with dots) is compared with the predicted trace by RNN (orange line with dots).

The neuromorphic vision system is also promising in the task of object tracking. Figure 4a schematically illustrates a flow chart of the object tracking. The box refers to the field of view defined by the retinomorphic sensor and the cross is the tracked target. The profile of the moving cross is extracted by the retinomorphic sensor and input into a recurrent neural network (RNN) as spatiotemporal features to enable the cross tracking. To demonstrate this proof of principle, we set a threshold current value for the retinomorphic sensor before measurement. At the beginning, the cross in the field of view is sensed and processed by the retinomorphic sensor. Then the processed cross is recognized by a trained memristive neural network. Afterwards, the coordinates of edge position are measured as output when the total current in the retinomorphic sensor exceeds the threshold value (middle panel of Fig. 4a). By considering all the positions of pixels in the edge of the cross, we obtain the averaged coordinates (X_n_, Y_n_) at a certain moment T_n_. To track the cross, the location information at a certain moment is input into an RNN for predicting the location of the moving cross at the next moment, which has already been used to process temporal data on the memristive crossbar [12,51,52].

We demonstrate cross tracking by networking the retinomorphic sensor with RNN. The RNN used for cross tracking includes two-input neurons, ten-hidden layer neurons and two-output neurons. We trained the RNN as below: the coordinates (X_n_, Y_n_) at T_n_ and H_n−1_ are fed into the hidden layer to generate C_n_ and H_n_ (as schematically illustrated in the middle panel of Fig. 4a), where C_n_ represents the coordinates predicted by RNN at T_n_ and H_n_ is the state vector at T_n_ generated from the previous state H_n−1_ in the hidden layer_._ The backpropagation through time algorithm was implemented for the RNN training, and the mean squared error was reduced to less than 10^−2^ after training 150 epochs (the details for the RNN training are provided in the Methods section). After training, the neuromorphic vision system is able to track the cross with good performance. Figure 4b compares the moving trajectory measured by the neuromorphic vision system and that predicted by the RNN. The good agreement between two traces indicates that the neuromorphic vision system is promising in object tracking, which is further supported by the real-time tracking video as shown in the Supplementary Movie.

## CONCLUSION

In summary, we, for the first time, realize a neuromorphic vision system by networking a retinomorphic sensor with a large-scale memristive crossbar. The sensor has been fabricated by using WSe_2_/h-BN/Al_2_O_3_ vdW heterostructure to emulate the function of retinal information processing. The 1T1R memristive crossbar in the networked system serves as the brain-inspired neural network for visual perception. With such a networked system, we demonstrate image recognition and object tracking, highlighting the potential application of image sensing, processing and recognition in the full analog regime. Our work indicates that we may envision promising applications of the neuromorphic vision system at the edge of the Internet of Things.

## METHODS

### Fabrication and measurement of phototransistor array

The bottom electrodes (Ti 2 nm/Au 30 nm) on the silicon substrate were patterned by a standard electron beam lithography (EBL) and lift-off process with 15 μm width. The Al_2_O_3_ are subsequently deposited by atom layer deposition (ALD) onto the bottom electrodes. WSe_2_ and h-BN flakes were mechanically exfoliated and transferred onto the Al_2_O_3_ layer to fabricate the vdW heterostructures, followed by an annealing process at 573 K in an argon atmosphere for 2 hours. We deposited Pd 5 nm/Au 45 nm onto the heterostructure as the source and drain electrodes respectively and annealed the fabricated devices again to remove resist residue. To confirm the thickness of materials used, we have used the atom force microscopy (AFM). All the fabricated vdW devices were then placed onto the designed printed circuit boards and interconnected to each other by using standard bonding techniques. The phototransistor array was then connected to our lab-made switching matrix box. A data acquisition card (National Instruments, PCIe-6351) and current amplifier (Stanford Research Systems, Model SR570) were used for current measurements. A source measurement unit (Keithley, 2636A) was used to apply gate voltages to the devices in the retinomorphic sensor.

### Image processing with the phototransistor array

To demonstrate image processing, we have used the 128 × 128 Lenna image. The image was segmented and converted into a sequence of 3 × 3 voltage signals by Python to drive a 3 × 3 laser array. The laser array was controlled by a multichannel relay and LabVIEW. Eventually, the measured data were rearranged in a sequence to construct the processed image by Python. All measurements were performed in a nitrogen atmosphere. The image of measurement system is provided in Supplementary Fig. 9.

### The analysis of the processed Lenna image

For the image processed by edge enhancement, we analyzed the original (Fig. 2c), experimental (Fig. 2d) and simulation (Supplementary Fig. 4) by Python to extract the grayscale of each pixel in these images and presented the counts distribution in the histogram with a Gaussian fit curve. For the stylization image, we first used Python to invert the original Lenna image to obtain a new image. Then the grayscale of each pixel on the diagonals of this image and other processed images (including experimental and simulation) were compared and normalized with respect to 255 as the operation error.

### Fabrication and training of a large-scale memristive crossbar

The large-scale memristor crossbars were integrated with transistor arrays via photo lithography, thin-film deposition and lift-off technology. We sputtered Ag/Pd as a metal vias, followed by a lift-off process and annealing of the samples at 573 K for 1.5 h. We sputtered a Pd/Ta adhesive layer as the bottom electrode. We deposited a 5 nm HfO_2_ switching layer using ALD. Photo lithography and reactive ion etch (RIE) were utilized to pattern the switching layer. Finally, we sputtered a thick Ta layer as the top electrode and thick Pd layer as the passivation layer, respectively.

We connected each memristor with a single transistor in series to mitigate the sneak-path current issue. To demonstrate brain-inspired visual perception, we networked the retinomorphic sensor with the memristive crossbar. We converted the current outputs of the retinomorphic sensor into voltage signals through a current-to-voltage converter and then transferred the voltage signals to input vectors matching the size of the memristive crossbar for training the ANN. The ANN contains a hidden layer with 37 neurons. In each layer of the ANN, we fed the input vectors into the crossbar through row lines and measured output vectors from column lines. Each weight value was represented by the difference in conductance between two memristors. Error backpropagation was conducted by reading out the conductance of the memristors and calculating the corresponding error in the computer. The cross-entropy loss function and root mean square propagation were chosen for weight update. A mini-batch size of 100 was used in the training process.

### Training of the recurrent neural network

We constructed a recurrent neural network containing two input-neurons, ten hidden-neurons and two output-neurons. By constantly training, the predicted trace would be close to the experimental trace. The goal of the training process was to minimize a loss function *L*, which is a function of the network output *y^t^* and the target $y{_{\mathit {target}}^t}$. We summed the mean square loss error over all time steps $L\ = \mathop \sum \nolimits_{p = 1}^P \mathop \sum \nolimits_{t = 1}^T \frac{1}{2}\| {y^t} - y{_\mathit {target}^t{\|}^2}$, for the prediction experiment, where *p* indexes over the sample. Specifically, the gradients were calculated using the backpropagation through time algorithm. The output of the last layer in the recurrent neural network and the output delta of the hidden layer were calculated through $\delta _y^t = g^{\prime}({{y^t} - y_{\mathit {target}}^t} )\ $and $\delta _h^t = \ f^{\prime}({(W{(\delta _y^t)^T} + U{( {\delta _h^{t + 1}} )^T})^T}$, respectively, where *g*^′^ and *f*^ ′^ are the derivative of the activation functions, *T* represents time interval, and *W* and *U* are the weight matrices for the input layer and hidden input layer of RNN, respectively.

## Supplementary Material

nwaa172_Supplement_File
