# Supplementary material for: Networking retinomorphic sensor with memristive crossbar for brain-inspired visual perception
Source: Natl Sci Rev. 2020 Jul 25;8(2):nwaa172. doi: 10.1093/nsr/nwaa172 (PMC8288371; doi:10.1093/nsr/nwaa172)
Supplement: nwaa172_Supplement_File [file nwaa172_supplement_file.zip › Supplementary_data.docx]

Supporting Information

**Networking retinomorphic sensor with memristive crossbar for brain-inspired visual perception**

*Shuang Wang, Chen-Yu Wang, Pengfei Wang, Cong Wang, Zhu-An Li, Chen Pan, Yitong Dai, Anyuan Gao, Chuan Liu, Jian Liu, Huafeng Yang, Xiaowei Liu, Bin Cheng, Kunji Chen, Zhenlin Wang, Kenji Watanabe, Takashi Taniguchi, Shi-Jun Liang^*^and Feng Miao^*^*

**
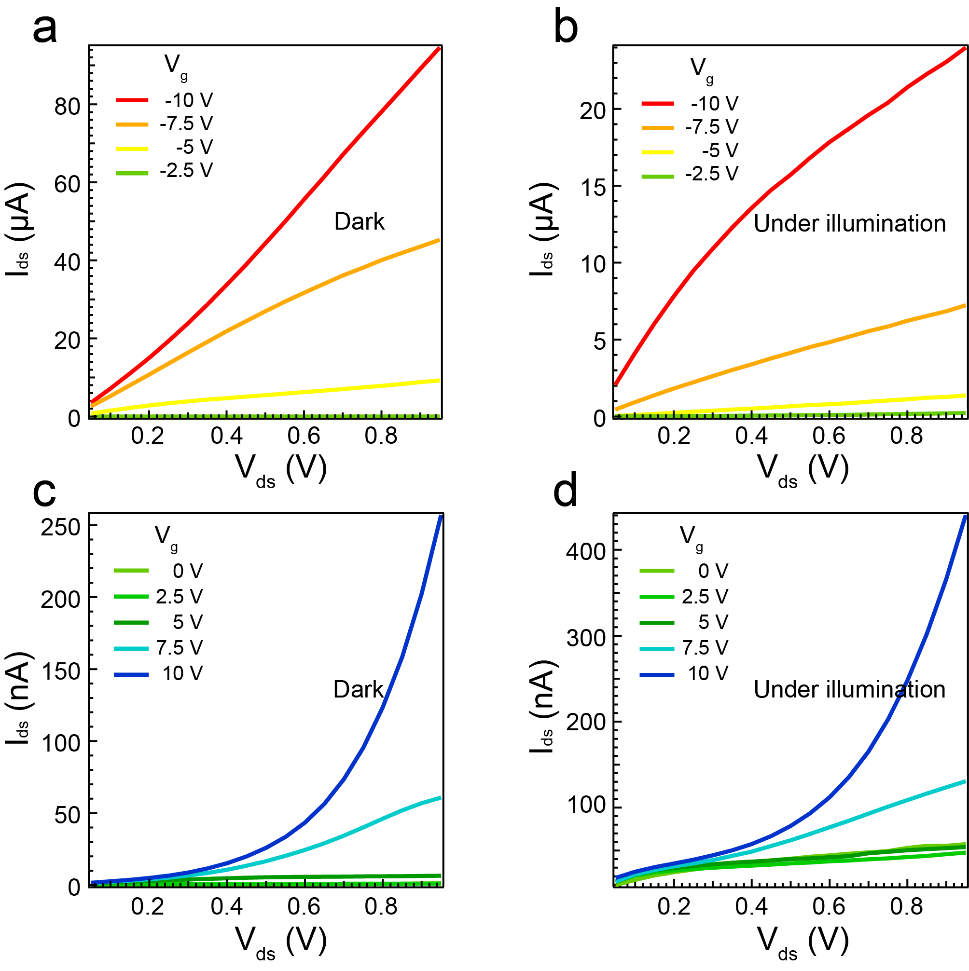
**

**Supplementary Fig. 1. Electrical characterization of the vdW device with and without light illumination.** (a-b) I-V curves with the condition of dark and light illumination for different negative gate voltages. The light illumination suppresses the current compared to that without light illumination. (c-d) I-V curves with the condition of dark and light illumination for different positive gate voltages. In this case, the light illumination leads to an increase in the current level under positive gate voltage.

**
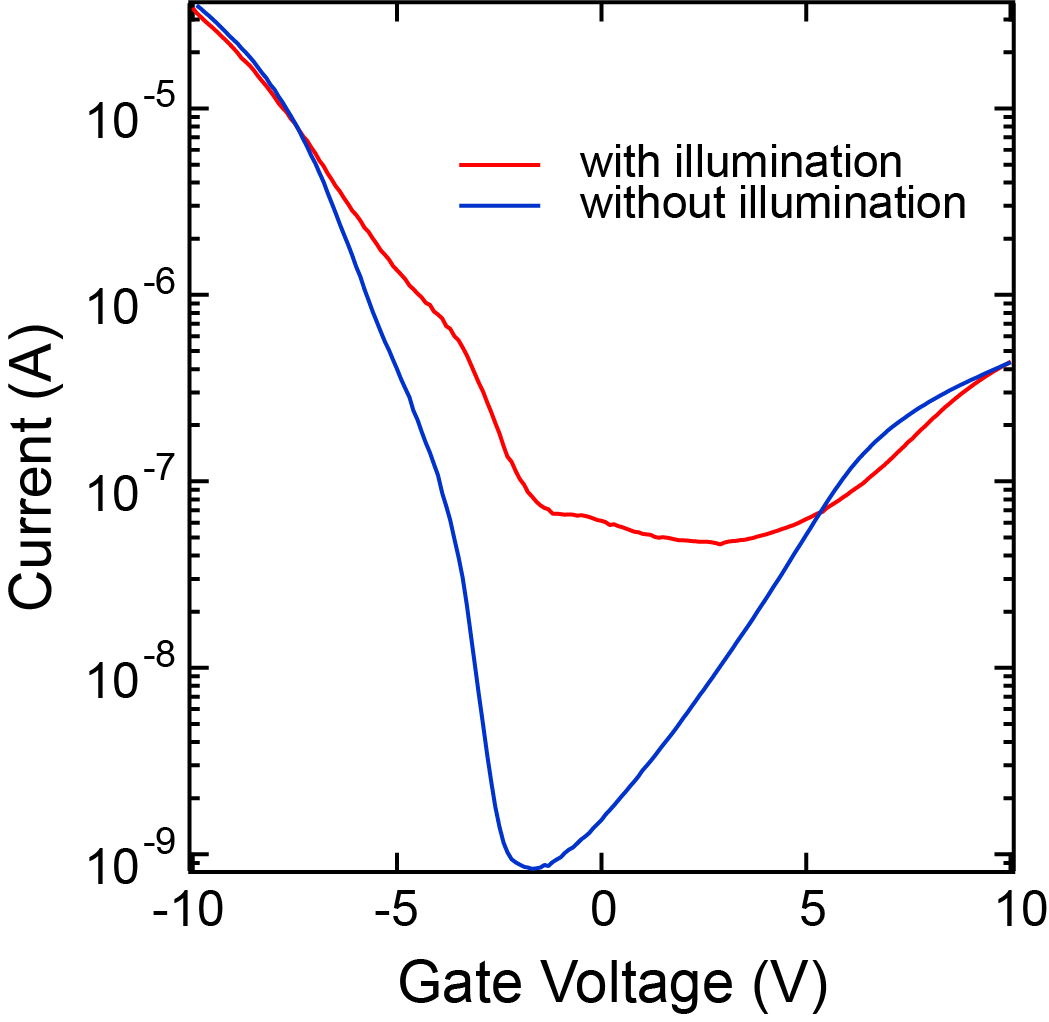
**

**Supplementary Fig. 2.** The transfer curves of vdW device with (red line) and without (blue line) light illumination.


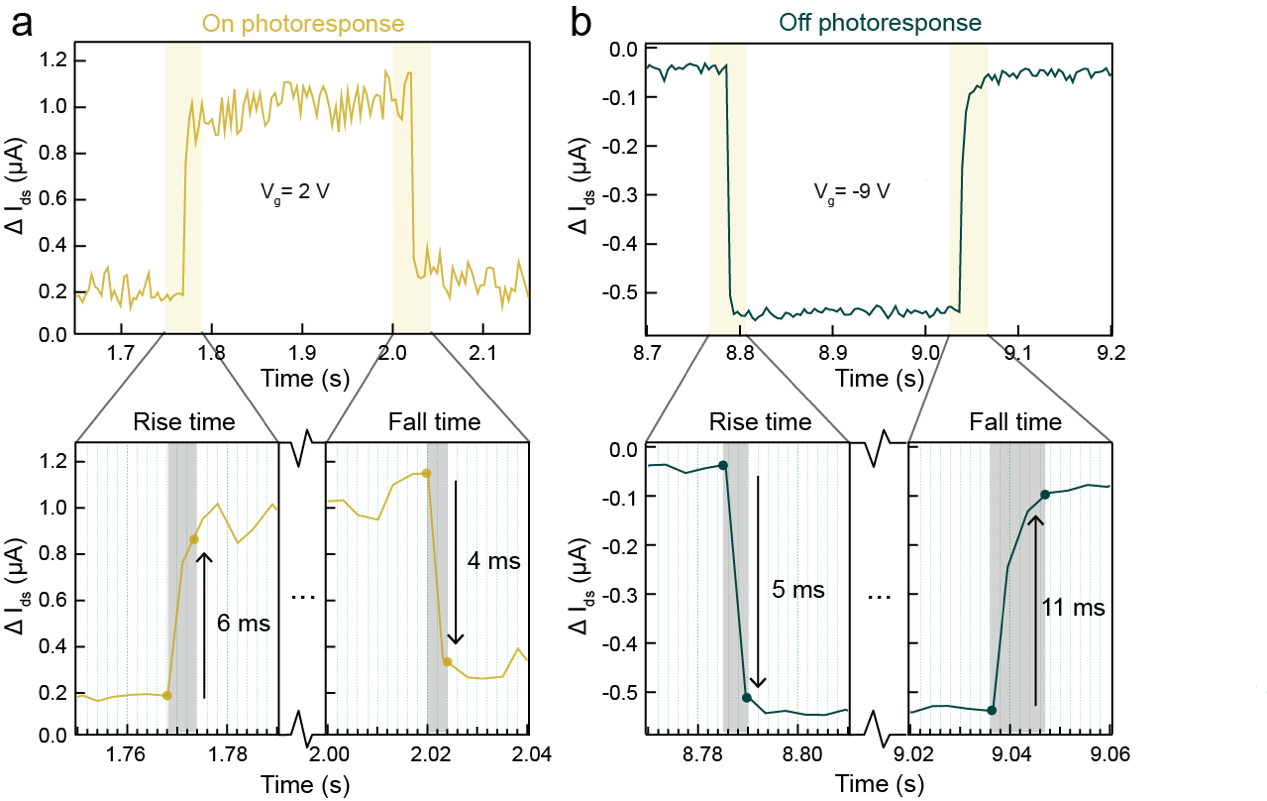


**Supplementary Fig. 3.**  Photoresponses of On and Off devices.

**
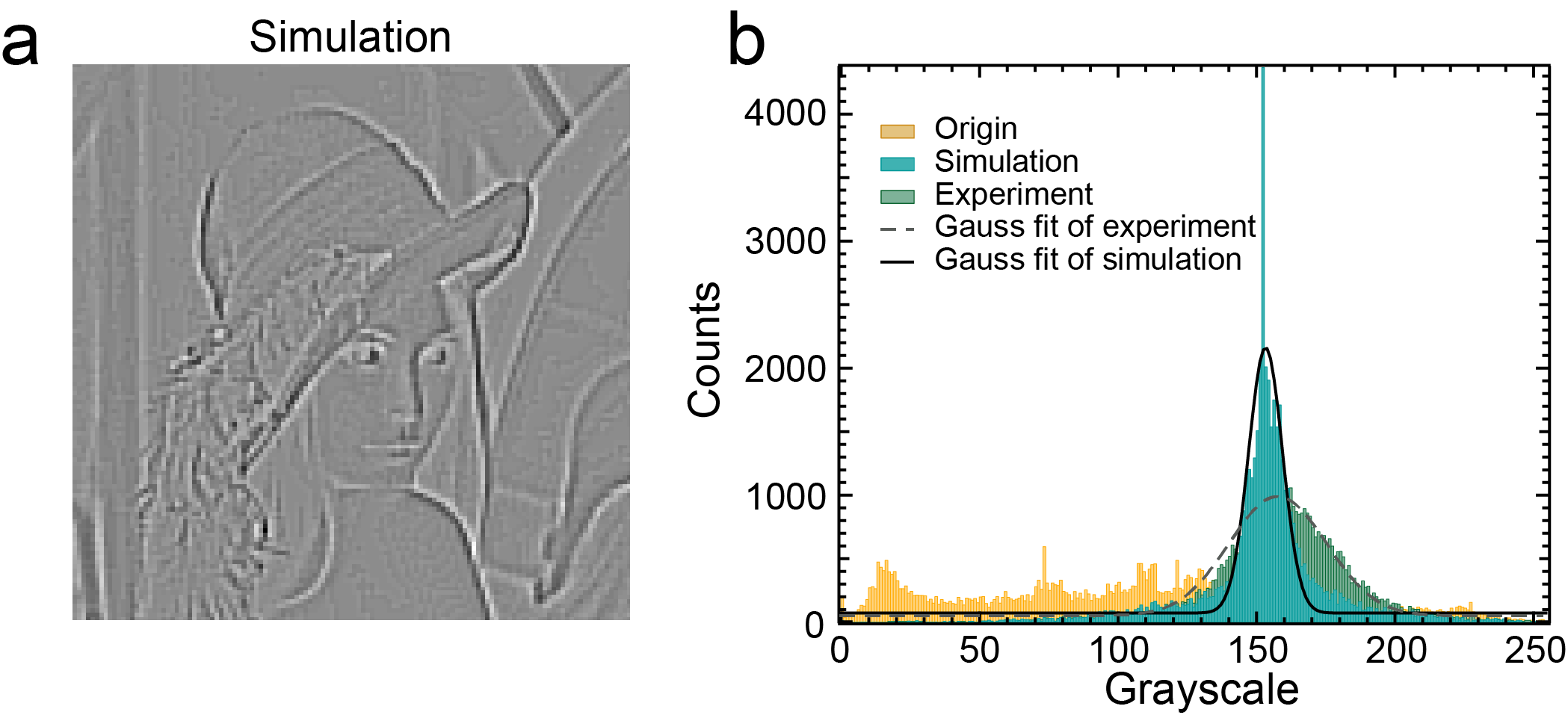
**

**Supplementary Fig. 4. Edge enhancement.** (a) The simulation image of the original Lenna image by of edge enhancement kernel. (b) Grayscale distribution of the origin (orange, Fig 2c), experiment (green, Fig 2d) and simulation image (blue), which are fitted with Gaussian function (dashed and solid lines). The experimental and simulation results give rise to similar behaviors of edge enhancement.

**
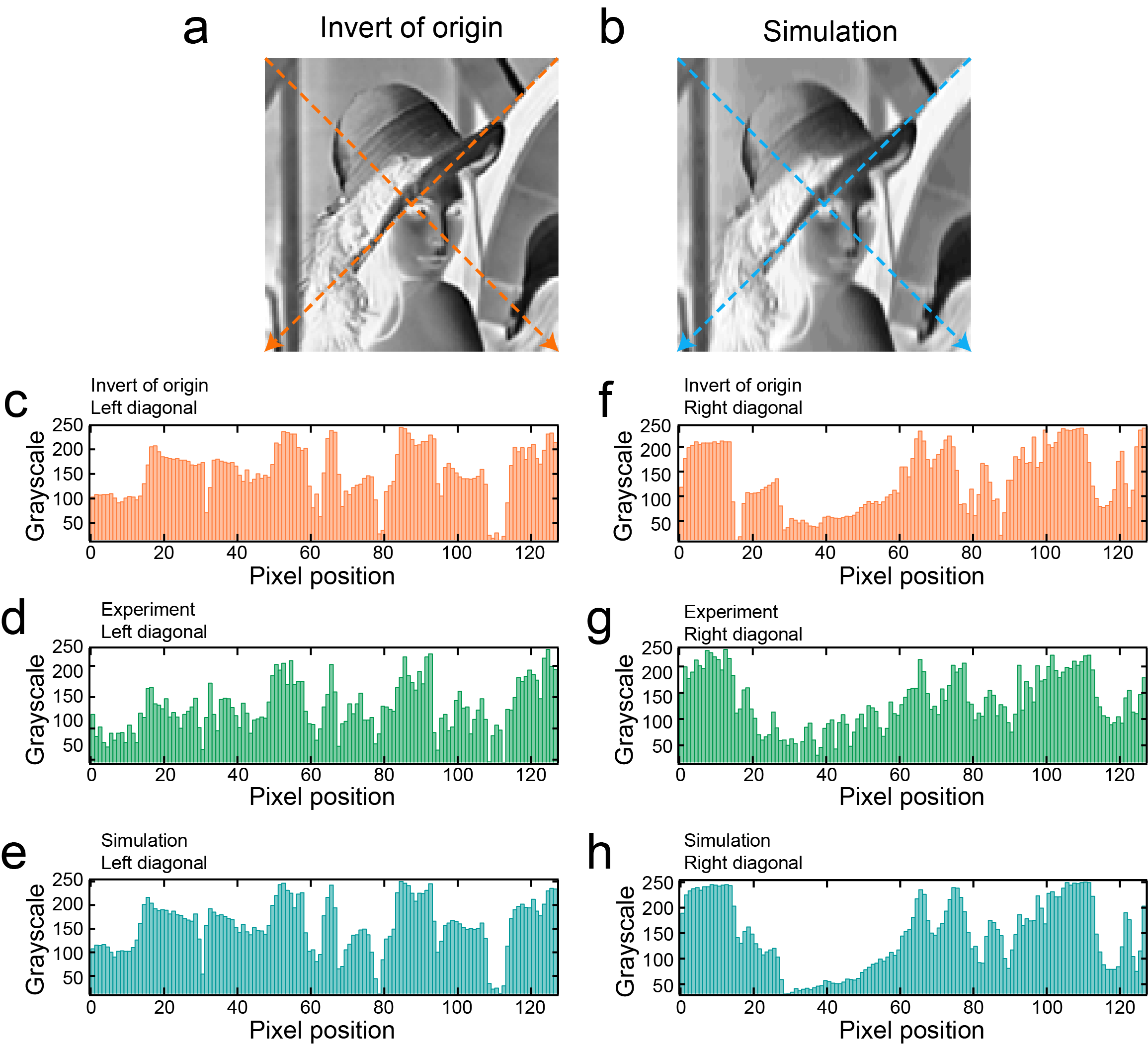
**

**Supplementary Fig. 5. Comparison between simulation and experimental results by stylization kernel** (a) The image with inverted grayscale of the origin Lenna image. (b) The simulation image by stylization kernel. (c-h) show the grayscale versus pixel positions along two diagonal directions of the corresponding images in (a), Figure 2f and (c).

**
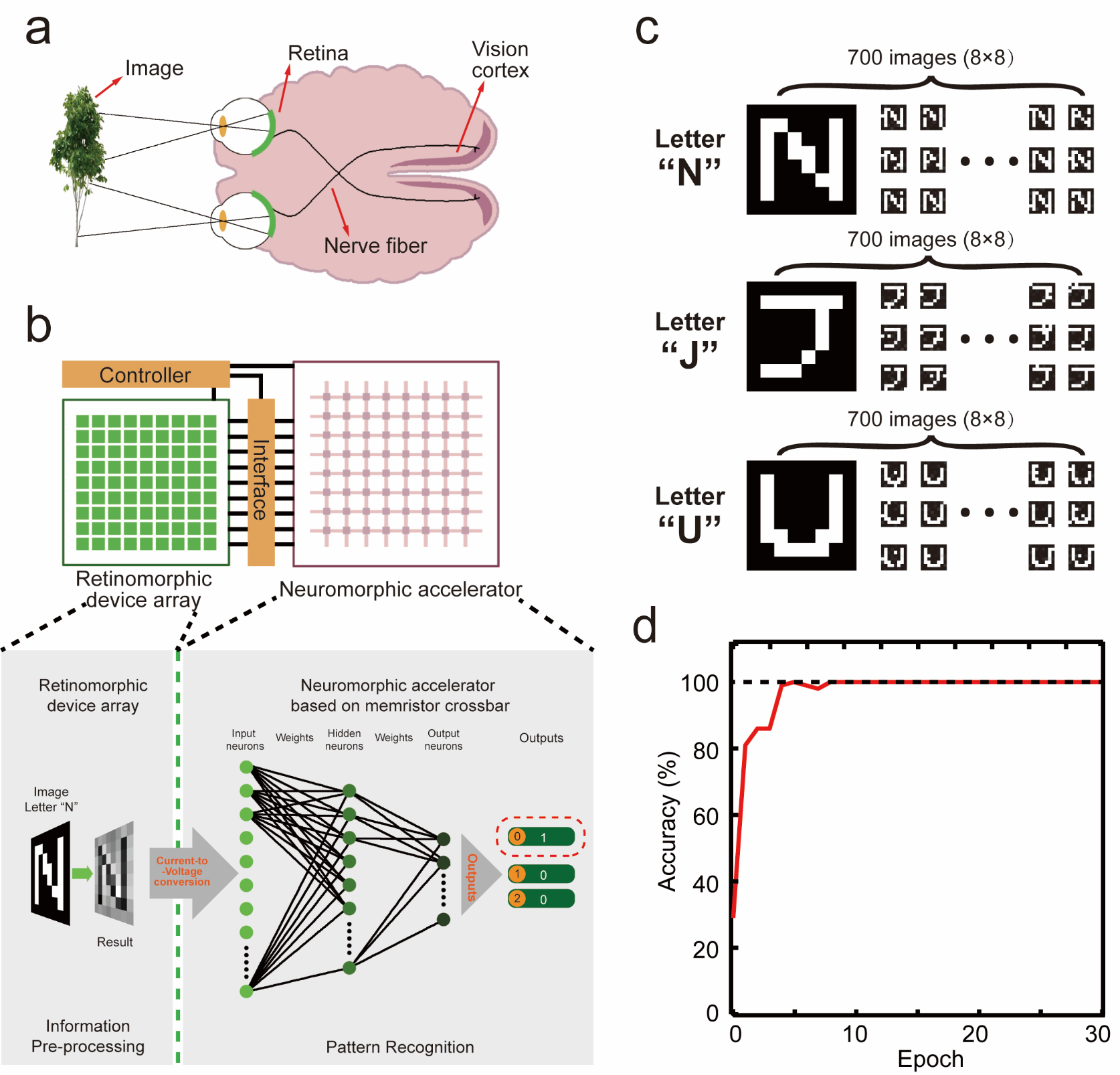
**

**Supplementary Fig. 6.** The derived 8×8 ‘N’, ‘J’, ‘U’ letters with embedded noise. The images were generated by Python.

**
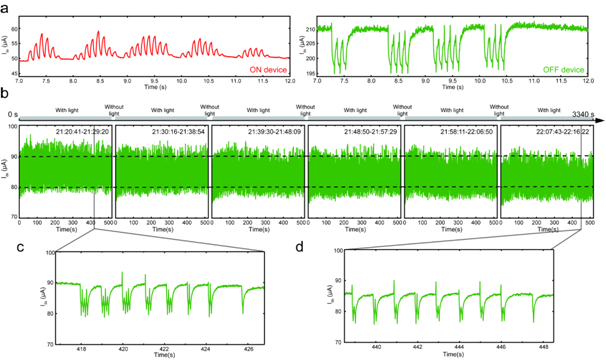
**

**Supplementary Fig. 7. MNIST dataset measurement and device endurance test.** (a) Original data of On and Off devices during MNIST dataset measurement. Light intensity changes over time according to the voltages sequence. Note that the photoresponse of On device was measured for different light intensities. (b) Endurance of Off device. (c) and (d) show photoresponse Off-device at the start and end of measurement. It is clear that the key photoresponse features of Off-device were retained during the MNIST dataset measurement.

**
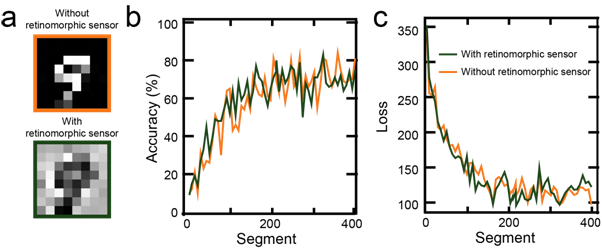
**

**Supplementary Fig. 8. The MNIST database recognition.** (a) The 8×8 image derived from MNIST database with (bottom panel, green square) and without (top panel, orange square) the early processing of the retinomorphic sensor. (b) The accuracy and (c) the loss under training of the two different configurations.


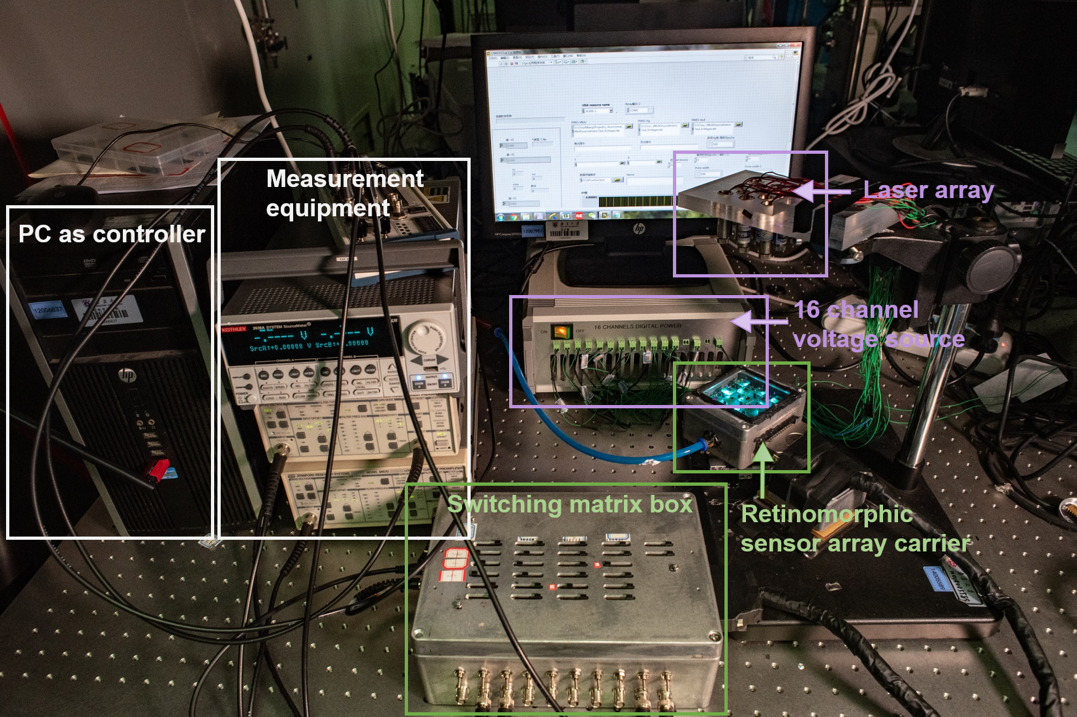


**Supplementary Fig. 9. Image of home-made measurement system**
